# Transient transfection of serum-free suspension HEK 293 cell culture for efficient production of human rFVIII

**DOI:** 10.1186/1472-6750-11-114

**Published:** 2011-11-24

**Authors:** Kamilla Swiech, Amine Kamen, Sven Ansorge, Yves Durocher, Virgínia Picanço-Castro, Elisa MS Russo-Carbolante, Mário SA Neto, Dimas T Covas

**Affiliations:** 1 Regional Blood Center of Ribeirão Preto, University of São Paulo (USP), Ribeirão Preto, Brazil; 2 Department of Pharmaceutical Sciences, Faculty of Pharmaceutical Sciences of Ribeirão Preto, University of São Paulo, Ribeirão Preto, Brazil; 3 National Research Council Canada, Biotechnology Research Institute, Montreal, Quebec, Canada; 4 Department of Clinical, Toxicological and Food Science Analysis, Faculty of Pharmaceutical Sciences of Ribeirão Preto, University of São Paulo, Ribeirão Preto, Brazil; 5 Department of Clinical Medicine, Faculty of Medicine of Ribeirrão Preto, University of São Paulo (USP), Ribeirão Preto, Brazil

## Abstract

**Background:**

Hemophilia A is a bleeding disorder caused by deficiency in coagulation factor VIII. Recombinant factor VIII (rFVIII) is an alternative to plasma-derived FVIII for the treatment of hemophilia A. However, commercial manufacturing of rFVIII products is inefficient and costly and is associated to high prices and product shortage, even in economically privileged countries. This situation may be solved by adopting more efficient production methods. Here, we evaluated the potential of transient transfection in producing rFVIII in serum-free suspension HEK 293 cell cultures and investigated the effects of different DNA concentration (0.4, 0.6 and 0.8 μg/10^6 ^cells) and repeated transfections done at 34° and 37°C.

**Results:**

We observed a decrease in cell growth when high DNA concentrations were used, but no significant differences in transfection efficiency and in the biological activity of the rFVIII were noticed. The best condition for rFVIII production was obtained with repeated transfections at 34°C using 0.4 μg DNA/10^6 ^cells through which almost 50 IU of active rFVIII was produced six days post-transfection.

**Conclusion:**

Serum-free suspension transient transfection is thus a viable option for high-yield-rFVIII production. Work is in progress to further optimize the process and validate its scalability.

## Background

Hemophilia A is a bleeding disorder caused by a deficient coagulation factor VIII (FVIII) that affects one in 5,000 to 10,000 males. Treatment for hemophilia A patients consists of replacement therapy using human plasma-derived FVIII (pdFVIII) or recombinant FVIII (rFVIII). Although effective, the pdFVIII replacement therapy has some drawbacks such as limited availability and the risk of transmitting blood-borne diseases to patients [1]. Recombinant factor VIII (rFVIII) emerges as an alternative to pdFVIII for the treatment of hemophilia A. However, efficient production of human rFVIII has been shown to be difficult [2-4]. The rFVIII expression levels are significantly lower than those of other recombinant proteins comparable in structure, size, and complexity [3]. Also, the expression is two or three orders of magnitude lower than other recombinant proteins produced with similar strategies [2]. This may be due to a low rFVIII mRNA level, a very inefficient transport of the primary translation product, and poor FVIII cellular secretion [5-7]. Some progress has been made towards recognizing the bottlenecks in FVIII biosynthesis, which primarily occur in the endoplasmic reticulum [8,9].

rFVIII is exclusively produced in cultured mammalian cells such as baby hamster kidney or Chinese hamster ovary cells, using large-scale bioreactors. Several techniques are used to maximize production. These include amplification of the FVIII transgene using dihydrofolate reductase/methotrexate selection, addition of FVIII stabilizing agents such as bovine/human albumin, or co-expression of the von Willebrand factor (vWf), and use of continuous-perfusion fermentation to maximize cell growth, cell density, and product recovery [4].

This has led to rather inefficient commercial rFVIII manufacturing and, in turn, important limitations concerning its production which is reflected in its high cost and low availability that even led to product shortage in economically privileged countries [10,11]. This situation may be solved by adopting more efficient and effective production methods [10].

Large-scale transient transfection is an efficient technology for the fast production of significant amounts of recombinant proteins [12,13] and it may represent an ingenious alternative for rFVIII production. Furthermore, scalable transient expression is an extremely effective technique for rapid screening of functional therapeutic candidate cell clones and subsequent manufacturing [13].

In this study we investigated, for the first time, the potential of transient transfection in producing rFVIII in serum-free suspension HEK 293 cell cultures. Through transfection using the polycationic polymer polyethylenimine (PEI) at high cell density, we were able to produce active rFVIII of up to 0.6 IU/mL within 4 days after transfection using a scalable system. After optimization of the production conditions, we were able to produce 49.3 IU of active rFVIII (120 mL of culture volume) within only 6 days after transfection.

## Methods

### Cell and Culture Media

A HEK293SF-3F6 cell line originally developed for the production of adenoviral vectors was used [14]. This clone has been selected for high-yield production in suspension and serum-free media. Additionally, a master cell bank is available for manufacturing of clinical material. The cell line presents great transfection potential and it grows in suspension under serum-free conditions. The cell line was cultured in HyQSFM4TransFx293 (HyQ) (Hyclone, Logan, UT, USA), a commercial medium specially developed for the transfection of HEK293 cells, supplemented with 5% Cell Boost 5 (CB5, Hyclone).

### Transient Transfection

The constructs used in this study were based on the replication-deficient bicistronic lentiviral vector cPPT-C(FVIIIdelB)IGWS, which codes for human B-domain deleted FVIII(FVIIIdelB), the deleted region of B domain was from from 2428 to 5067 nucleotides (full-length FVIII-cDNA accession number K01740) and this vector has the IRES element followed by the enhanced green fluorescent protein (EGFP). Our lentiviral vector has the CMV promoter, cPPT (central Polypurine Tract) from the HIV-1 integrase gene that increases the copy number of lentivirus integrating into the host genome, thus increasing viral titer and also the WPRE (Woodchuck Posttranscriptional Regulatory Element) from the woodchuck hepatitis virus that increases transgene expression. A more detailed vector description can be found in Tonn et al. [15].

The transfection protocol has been published previously [16]. Pre-cultures underwent passages every 2-3 days to keep the cells in their exponential growth phase. A few hours before transfection, the cell suspension was centrifuged (300 *g *for 5 min) and re-suspended in fresh medium at 5 × 10^6 ^cell/mL.

Because of the instability of rFVIII, we conducted daily harvests and completed medium exchange by centrifugation (300 ***g ***for 5 min), starting 1 day post-transfection, in 125 mL Erlenmeyer flasks containing 20 mL of working volume. rFVIII-containing harvests were filtered through 0.45 μm HT Tuffryn membranes (Pall, Ann Arbor, MI, USA) to remove cell debris and were stored at -80°C until further analyses.

To determine the best transfection parameters for rFVIII production, three different DNA concentrations (0.4, 0.6, and 0.8 μg DNA/10^6 ^cells) were tested at 37°C. The potential of repeated transfections at three different times (0, 48, and 96 hours post-inoculation) and two different temperatures (34 and 37°C) were also tested. In the repeated transfection experiments, a new amount of polyplexes was added at 48 and 96 hours, besides the amount added in the beginning of the experiment. In this case, the amount of polyplexes was corrected based on the cell density determined in each culture time.

### Analytical Methods

Hemacytometer counts using erythrosine B dye exclusion were used to assess cellular density and viability.

Glucose, lactate, and ammonia concentration assessment were performed using Biolyser Analyser (Kodak, New Haven, CT). Amino acid concentration in fresh media and supernatants were quantified by HPLC (Waters Alliance System, Waters Corp., Milford, MA) using a modification of the Waters AccQ-Tag™ method as described by Cohen (2000).

The commercial ELISA kit Asserachrom FVIII:C Ag (Diagnostica Stago) was used to quantify the rFVIII production. To determine the rFVIII activity, we used the chromogenic kit COAMATIC^® ^Factor FVIII (Chromogenix, Instrumentation Laboratory SpA). Both kits were used according to manufacturer's instructions.

## Results

### Effect of DNA Concentration

**Figure 1:**
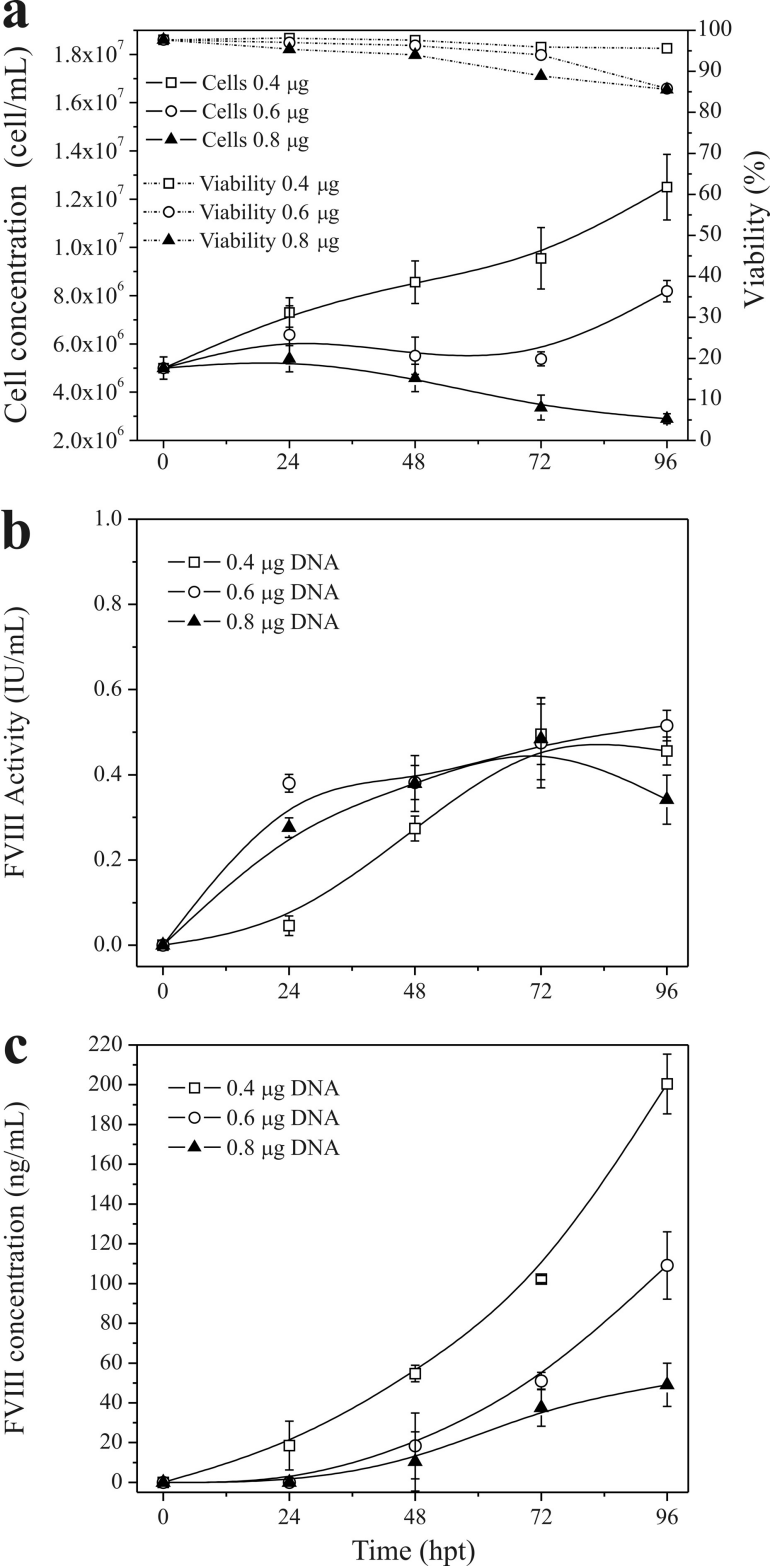
**High-density transfection of Hek293 cells**. Cells were re-suspended in 20 mL of fresh HyQSFM4TransFx293 medium to a final density of 5 × 10^6 ^cells/mL and transfected with 2, 3, and 4 μg of DNA and 4, 6, and 8 μg of PEI, respectively, in 125 mL Erlenmeyer flask and 37°C. a: total cell density and viability; b: rFVIII activity measured by chromogenic assay; and c: rFVIII measured by ELISA. Values shown are the averages (mean ± SEM) of the duplicated samples from one representative experiment.

For an initial evaluation of the potential of transient transfection on rFVIII protein production, transfections using different DNA concentrations were performed. High-density HEK293 cell cultures (5 × 10^6 ^cells/mL) were transfected with 0.4, 0.6, and 0.8 μg DNA/10^6 ^cells at PEI:DNA ratio of 2:1. Figure 1A shows that an increased concentration of PEI:DNA complex (polyplex) inhibits cell growth. Although cell viability did not decrease significantly when using DNA concentrations of 0.6 and 0.8 μg DNA/10^6 ^cells, we observed a large amount of cellular debris. After 96 hours of culture, the transfection efficiency was approximately 50% for all DNA concentrations and conditions tested (Table 1).

**Table 1:** Effect of transfection conditions on transfection efficiency, cumulative rFVIII production, and cell specific rFVIII production (qFVIII) rate by transient transfection of HEK293 suspension cultures

Condition	Transfection efficiency (%)	*Cumulative active rFVIII production (IU)	q_FVIII_ (ng FVIII/10^6 ^cell.h)
**0.4 μg DNA/10^6 ^cel**	51.1	25.4	0.24

**0.6 μg DNA/10^6 ^cel**	50.0	35.1	0.30

**0.8 μg DNA/10^6 ^cel**	55.9	29.6	0.21

**0.4 μg DNA/10^6 ^cel** **37°C Repeated Transfection**	57.3	21.9	0.43

**0.4 μg DNA/10^6 ^cel** **34°C Repeated Transfection**	52.8	49.3	0.58

The transfections were done using the PEI:DNA ratio of 2:1. *rFVIII cumulative production in IU was calculated using biological activity results.

The biological activity of the rFVIII produced using DNA concentrations of 0.6 and 0.8 μg DNA/10^6 ^cells was similar but slightly higher than the one obtained using 0.4 μg DNA/10^6 ^cells in the first 2 days post-transfection (Figure 1B). A larger difference was observed by ELISA quantification (Figure 1C). After 4 days of transfection, 200, 109, and 49 ng/mL of rFVIII were produced with DNA concentrations of 0.4, 0.6, and 0.8 μg DNA/10^6 ^cells, respectively. The DNA concentration of 0.6  μg DNA/10^6 ^cells produced the highest amount of cumulative active rFVIII (35.1 IU) when comparing with 0.4 and 0.8 μg DNA/10^6 ^cells. The cell-specific rFVIII production rate was also higher using 0.6  μg DNA/10^6 ^cells for single transfections, as shown in Table 1.

To apply a repeated transfection strategy, we hypothesized that the "quality" of the culture, i.e. a high number of viable cells in a good physiological state is a critical parameter; we thus decided to use the concentration of 0.4 μg DNA/10^6 ^cells in the following experiments. Despite the lower rFVIII specific activity obtained in this condition, the cell viability remained high, no cellular debris was observed and a higher amount of protein was quantified by ELISA while consuming the lowest amount of DNA.

### Effect of Temperature and Repeated Transfection

**Figure 2:**
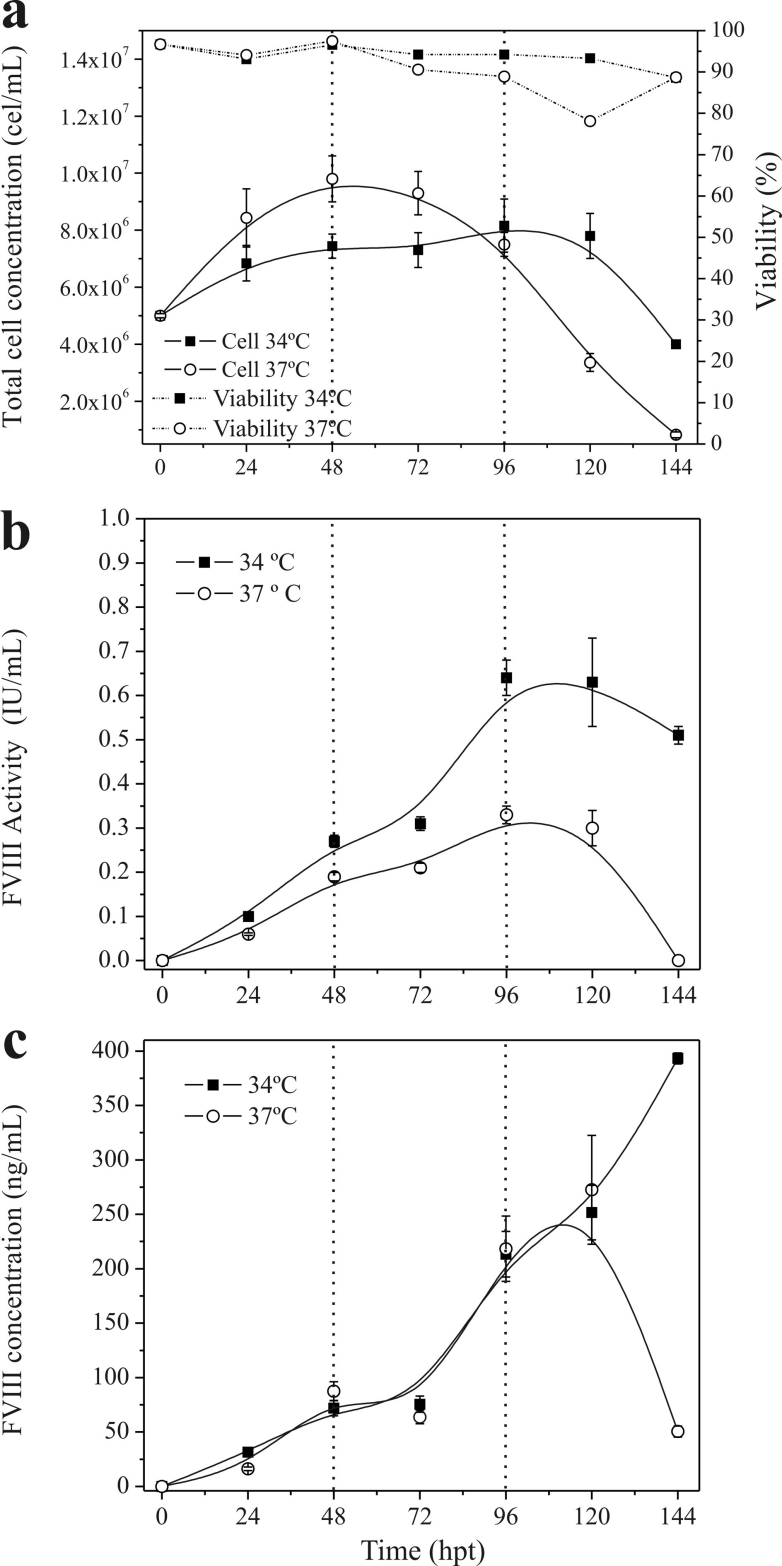
**High-density transfection of Hek293 cells**. Cells were re-suspended in 20 mL of fresh HyQSFM4TransFx293 medium to a final density of 5 × 10^6 ^cells/mL and transfected with 2 μg DNA and 4 μg PEI in 125 mL Erlenmeyer flask at 34°C and 37°C. Transfections were done at 0, 48, and 96 hours post-inoculation. a: total cell density and viability; b: rFVIII activity measured by chromogenic assay; and c: rFVIII concentration measured by ELISA. Values shown are the averages (mean ± SEM) of the duplicated samples from one representative experiment. The dotted lines indicate when the re-transfections were done.

The potential of repeated transfections at low temperature was also evaluated. Figure 2A shows the cellular growth and rFVIII production at 34°C and 37°C. Transient transfections were done at 0, 48, and 96 hours after inoculation. At 34°C, no cellular growth was observed but cells remained viable, even after re-transfecting them. The repeated transfection at 37°C had an inhibitory effect on cell growth. After the second round of transfection (48 hours after inoculation) a large amount of cellular debris was observed, and total cell concentration declined significantly after 72 hours (Figure 2A). The percentage of GFP-positive cells was measured at 48, 72, 96, and 120 hours after cell inoculation and remained in the range of 50-60% for both temperatures tested (34°C and 37°C).

Interestingly, the rFVIII produced at 34°C presented a higher biological activity than that observed at 37°C. At this temperature we were able to produce 0.64 IU/mL of active rFVIII 96 hours after inoculation through two rounds of transfections. In contrast, production at 37°C resulted in approximately 2-fold decrease in active rFVIII (0.33 IU/mL). Nevertheless, the kinetics for total rFVIII production (determined by ELISA) was similar. A significant difference between the two production temperatures tested was observed at 144 hours (393 ng/mL at 34°C against 50.5 ng/mL at 37°C).

Consequently, the cell-specific rFVIII production rate was 26% higher at 34°C when compared to production at 37°C (0.58 ngFVIII/10^6^cell.h against 0.43 ngFVIII/10^6^cell.h) (Table 1). After 144 hours of transfection, 49.3 IU of active rFVIII at 34°C and 21.9 IU at 37°C were produced.

**Figure 3:**
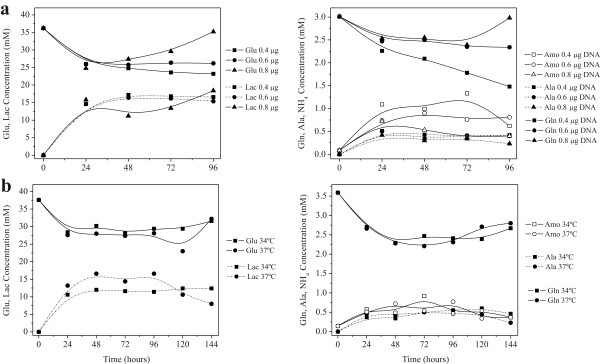
**Profile of nutrient consumption and by-product formation during high-density transfection of Hek293 cells**. Cells were re-suspended in 20 mL of fresh HyQSFM4TransFx293 medium to a final density of 5 × 10^6 ^cells/mL and transfected using a PEI:DNA ratio of 2:1. (A) Transfection using 0.4, 0.6, and 0.8 μgDNA/10^6 ^cells at 37°C; (B) Repeated transfection using 0.4 μgDNA/10^6 ^cells at 0, 48, and 96 hours post-inoculation at 37°C and 34°C.

**Figure 4:**
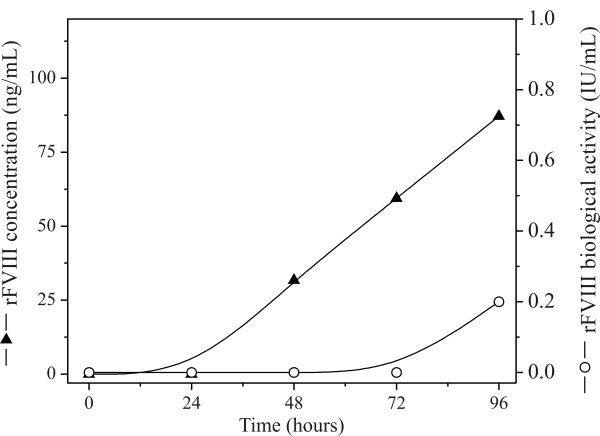
**High-density transfection of Hek293 cells in batch mode (without changing the medium)**. Cells were re-suspended in 20 mL of fresh HyQSFM4TransFx293 medium to a final density of 5 × 10^6 ^cells/mL and transfected with 2 μg DNA and 4 μg PEI in 125 mL Erlenmeyer flask at 37°C.

Complete replacement of the culture medium simulated a perfusion operation and prevented nutrient exhaustion and accumulation of inhibitory by-products (Figure 3). Even with high cell concentrations we did not observe the depletion of glucose and glutamine or the formation of toxic by-products that could potentially inhibit cell growth. Nevertheless, complete replacement of the culture medium provides a higher production of active FVIII as can be seen by comparing the levels obtained in batch mode (Figure 4) with those obtained in Figures 1 and 2. When the medium was not changed, the expression of rFVIII was lower than what was achieved through simulation of perfusion mode operation (up to 400 ng/mL or 0.6 IU/mL). In batch mode, no significant rFVIII biological activity was detected until 96 hours post-transfection but even then the levels were much lower (0.2 IU/mL).

## Discussion

In this study, we evaluated the potential of the transient transfection technique for rFVIII production. More specifically, we investigated the effect of different DNA concentration and repeated transfection at 34° and 37°C on rFVIII production by human HEK293 cells cultured in serum-free suspension.

The transfection efficiency largely depends on the formulation of transfection complexes, which usually represents 10% of the culture volume. The cell number-based DNA dosage and the DNA:PEI mass ratio are the most important variables affecting transfection efficiency [17]. A PEI:DNA mass ratio of 2:1 is considered the optimal ratio for transient transfection of HEK293 cells [12,18], which is the same cell line than the one used in our study. Therefore, we kept this PEI:DNA ratio when testing different DNA concentrations for rFVIII protein production.

For higher polyplex amounts, we observed a decrease in cell growth. Nevertheless, no significant differences in transfection efficiency and in the biological activity of the rFVIII were noticed. It has been suggested that lower amounts of polyplexes minimize toxic effects of PEI and also minimize cell aggregation, leading to higher productivity after transient transfection [19]. Besides, for a repeated transfection approach it is very important to maintain a high viable cell concentration during the whole culturing period. When the DNA concentration of 0.8 μg/10^6 ^cells was used, the cell viability started to decrease at 48 hpt (hours post transfection) and reached low values at 96 hpt. We thus hypothesized that it would not be productive to retransfect this culture. Thus, we applied a DNA concentration of 0.4 μg/10^6 ^cells, which presented a satisfactory cell growth and viability, as the standard condition. This DNA concentration has also the advantage of reduced costs.

The production of rFVIII and the maintenance of its biological activity are very difficult, especially in serum-free medium. Whereas the expression of rFVIII in serum containing medium, which includes vWF (von Willebrand Factor) that acts as a natural FVIII stabilizer, usually results in high yields, the expression of this complex protein in serum free medium imposes further challenges due to the absence of vWF [3]. There is evidence showing that under serum free conditions, rFVIII binds to the phospholipids on the cell membrane resulting in degradation [20]. Therefore, to protect, stabilize and release rFVIII into the culture medium, the coexpression with vWF has been performed [3]. In another approach to solve this problem, Winge [21] shows that large amounts of active rFVIII could be released from serum-free recombinant HEK293 cells through exposure to a solution of high ionic strength. We thus hypothesized that there is a link between these observations and what was found concerning the similar biological activity of rFVIII in the experiments with different DNA concentrations. Although a higher amount of protein had been produced using 0.4 μg DNA/10^6 ^cells, the biological activity remained at a similar level; this could have been due to a superior level of cell membrane binding and consequent degradation due to the increased cell concentrations. Strategies to overcome these challenges will be further developed.

The best condition for rFVIII production was obtained with repeated transfections at 34°C (Table 1). After 144 hours of culture, almost 50 IU of active rFVIII was produced. If all the rFVIII protein produced at 34°C (approximately 400 ng/mL) was active, a biological activity of 2 IU/mL and a production of 100 IU of rFVIII could potentially be achieved. This condition also resulted in the highest cell-specific rFVIII production rate.

Cells cultured below physiological temperatures have genetic, molecular, and phenotypic alterations [22]. Cells cultured under mild hypothermic conditions (30°C to 35°C) have been found to induce an actively regulated growth reduction in cells within the S or G1 phase of the cell cycle [23] and an increased rate of recombinant protein synthesis. Actively metabolizing cells maintain their growth arrest through inhibiting growth mechanisms. In addition to an increase in productivity induced by growth arrest, cultivation of cells under mild hypothermic conditions offers other relevant advantages: extended culture times (lower cell populations reduce overall nutrient uptake and waste production) [24], decreased O_2 _demand [25], reduced intermolecular product aggregation [26], increased sensitivity to pH changes [27,28], and a decreased sensitivity to pro-apoptotic agents [29,30]. Protein sialylation [24], acidic glycoforms [31], and antennary structures [31] are relevant quality parameters that are improved in cells cultured under hypothermic conditions. Beyond to providing a good nutritional environment for cell growth, complete replacement of the culture medium provided an adequate culture environment for FVIII expression and maintenance of biological activity. This finding is not surprising since, the bioprocesses currently used for commercial production of rFVIII are based on continuous perfusion or fed-batch bioreactor cultures.

Comparing the rFVIII levels produced in this work with results reported by other groups is difficult because most studies provide information on the expression levels in cell basis (IU/10^6 ^cells/24 hour) and no information is provided on the volumetric concentration and on the kinetic expression profile, which are needed when analyzing the scalability of the process. Furthermore, the expression is always performed in static cultures and sometimes in serum-containing medium. The approaches used to improve rFVIII production focus on identifying new host cell lines and expression vectors/promoters to enhance rFVIII expression [15,32-37] or engineering of the FVIII molecule to prolong its half-life [38-40]. Other efforts include the development of new recombinant cell lines and vectors for cell and gene therapy [41-45].

Recently, Spencer et al. [4] showed some kinetic data on porcine rFVIII expression in BHK cells while working with static cultures. Haack et al. [32] performed transient transfection of full-length and B-deleted rFVIII production in HEK293, COS, and CHO cells. The authors presented a kinetic expression profile but the levels reached were low (around 8-10 ng/mL). Chen et al. [33] studied the rFVIII transient expression in different cell lines (SMMC-7721, A549, Cos-7 and CHO) and found rFVIII levels in the range of 24 to 201 ng/mL. The highest expression level was obtained in the CHO cells. In this work, expression levels were 40 to 50-fold higher (400 ng/mL) than obtained by Haack et al. [32] and 2-fold higher than Chen et al. [33].

Previous work describing the transient expression of rFVIII reported lower yields (see above) and also used exclusively adherent cells in combination with costly transfection agents [32,33]. In contrast, suspension-grown HEK293 cells in combination with PEI-mediated transfection were used in this work. Whereas the yield obtained with adherent cells is generally limited by the cell culture surface area, suspension-grown cell lines show better and more straightforward scalability. Their use allows for production at high cell density in shake flask and continuous bioreactor perfusion cultures to reach high yields and rescue labile products in the cell free supernatant. For the development of an efficient and effective production process, the scale-up should ideally target an increased culture volume while achieving an enhanced volumetric productivity (such as attained here through production in high cell density suspension cultures); in contrast, only a higher number of production vessels or a larger surface area, as often done for adherent cultures (from T-flasks to roller bottles and cell factories), represents a sub-optimal scale-up. We have previously shown that transient transfection based processes can be successfully scaled up to 3 L stirred tank reactors using perfusion bioreactor operation [19]. We thus anticipate that scaling up rFVIII production from small scale medium replacement to continuous perfusion operation using an acoustic cell filter would be straightforward and should result in comparable protein yields.

Currently, there are no FDA-approved recombinant proteins generated by large-scale transient transfection. Several issues need to be addressed before the repeated transient transfection method can be implemented in a manufacturing environment. For example, batch to batch product consistency and process robustness remain to be demonstrated at large scale [46]. Other concerns comprise the cost-effective generation of sufficient amounts of plasmid DNA [13]. Although it seems to remain to some extent unclear what quality attributes such DNA would need to fulfill the use in commercial manufacturing, the challenges concerning high-yield plasmid DNA production for large scale transient transfection have been extensively addressed in the literature. For example, these include the removal of *E. coli *DNA and endotoxins [47-50]. In agreement with Geisse [51], the generation of recombinant DNAs for large scale applications should not be limited by current standard *E. coli *expression and purification techniques. Usually, from 2 to 3 L of bacterial cultures, 10-20 mg of plasmid DNA can be obtained. At commercial scale, this process can be easily adapated to bioreactor production to further improve the productivity. Indeed, Cheng et al reported the production of 1.5 g plasmid DNA from 3 L fermentation broth of *E. coli *in a cost effective manner, suitable for scaling up to meet the large demand of DNA [49]. All of these issues then need to be evaluated and weighed against the laborious and time-consuming generation of stable cell lines which could eventually lead to improved process yields. Overall, we do need to highlight that the ever-increasing number of publications on transient transfection technologies employed for recombinant protein production reflects the success of this approach in the past decade [51].

## Conclusion

To our knowledge this is the first study describing a rFVIII production method based on transient transfection in suspension serum-free cultures that can be operated at large scale. This method can be used to easily produce larger amounts of protein in a short period of time to be further used in functional characterization and pre-clinical studies. Work is in progress to further optimize the process and demonstrate its scalability.

## Competing interests

The authors declare that they have no competing interests.

## Authors' contributions

KS and SA designed and executed the experimental work. SA, VPC, EMSRC, MSAN participated in vector production. KS, AK, SA, YD, VPC, EMSRC, MSAN and DTC were involved in result interpretation and manuscript preparation. AK and DTC participated in the study design and coordination. All authors read and approved the final manuscript.
